# Characterization of antimicrobial activity of three *Lactobacillus plantarum* strains isolated from Chinese traditional dairy food

**DOI:** 10.1002/fsn3.1025

**Published:** 2019-04-29

**Authors:** Chang‐Hui Hu, Li‐Quan Ren, Ying Zhou, Bang‐Ce Ye

**Affiliations:** ^1^ Department of Food Science and Technology, School of Bioengineering East China University of Science and Technology Shanghai China; ^2^ Lab of Biosystems and Microanalysis, State Key Laboratory of Bioreactor Engineering East China University of Science and Technology Shanghai China; ^3^ School of Chemistry and Chemical Engineering Shihezi University Xinjiang China

**Keywords:** food additive, food preservation, *Lactobacillus plantarum*, organic acid, probiotic

## Abstract

Many *Lactobacillus plantarum* strains can secrete some antimicrobial substances and be added to food as antimicrobial agents and preservatives. In this study, three *L. plantarum* strains (P1, S11, and M7) with strong antimicrobial activity against three pathogenic bacteria were isolated from Xinjiang traditional dairy products. Five common organic acids produced by fermentation of strains play a key role in inhibiting three pathogenic bacteria. At the same pH, the antimicrobial activity of the fermentation broth against *Escherichia coli* and *Salmonella* is stronger than that of the organic acid alone. Thus, three kinds of antimicrobial agents (P1‐1, M7‐1, and S11‐1) mixed with five common organic acids were produced. Moreover, the antimicrobial activity against *Salmonella* ASI.1174 of the antimicrobial agents was about 30% higher than that of the fermentation broth. In addition, organic acid antimicrobial agents combined in different proportions can inhibit different pathogenic bacteria. According to this result, it is a potential approach to develop novel antimicrobial agents used in food preservation by mixing different organic acids.

## 1 INTRODUCTION

Lactobacilli are widespread microorganisms which have numerous applications in both industry and human health, including food preservation and probiotics (Altay, Karbancıoglu‐Güler, Daskaya‐Dikmen, & Heperkan, 2013; Cebeci & Gürakan, 2003). Among many *Lactobacillus* strains, *Lactobacillus plantarum* is a functional and important probiotic which can be found in many fermented foods, probiotic foods, and natural foods (Guidone et al., 2014). In recent years, some *L. plantarum* strains with unique functions such as *L. plantarum* CCFM8610, *L. plantarum* C88, *L. plantarum* strain 21B were discovered (Huang et al., 2017; Lavermicocca et al., 2000; Zhai et al., 2014). In addition, many *Lactobacillus* strains with strong antimicrobial activity had also been screened. *L. plantarum* D1 and *L. plantarum* D2 show significant inhibitory activity against *Salmonella* (Teneva, Denkova, Goranov, Denkova, & Kostov, 2017). *Lactobacillus acidophilus* A2, *L. acidophilus* Ac, etc., can inhibit *Candida albicans* NBIMCC 74 by cocultivation (Denkova, Yanakieva, Denkova, Nikolova, & Radeva, 2013). Many traditional fermented foods are a rich bacterial library for screening *L. plantarum* with antimicrobial activities. Ethnic minorities such as the Kazak and Mongolian people in the Xinjiang region of China have kept the habit of producing and eating fermented dairy products since ancient time. After thousands of years of domestication, these traditional dairy products retain many of the lactic acid bacteria that assist in the unique flavor of dairy products.

The conversion of sugar to lactic acid is still the main function of *L. plantarum*. In addition, production of exopolysaccharides, antimicrobial peptide, and specific beneficial compounds that are beneficial to the human body such as vitamins are other important properties (de Vries, Vaughan, Kleerebezem, & de Vos, 2006; Li, Gu, Yang, Yu, & Wang, 2017). Furthermore, *L. plantarum* strains can produce sundry antimicrobial compounds such as organic acids (primarily lactic and acetic acid), hydrogen peroxide, and antimicrobial peptides (Denkova, 2017; Lavermicocca et al., 2000; Nealmckinney et al., 2012). What's more, some studies have found that lactic acid bacteria biofilms show the capability to influence the survival and the multiplication of the pathogen (Guerrieri et al., 2009). Concurrently, the increasing attention of consumers for natural food urged scientific research to investigate the application of natural compounds in food to replace synthetic chemicals and additives as preservatives (Castro, Palavecino, Herman, Garro, & Campos, 2011). According to production of antimicrobial substances and harmless characteristics, *L. plantarum* could be the suitable candidates for natural antimicrobial agent (da Silva Sabo, Vitolo, González, & Oliveira, 2014). In recent years, many *L. plantarum* strains with the ability to inhibit pathogenic bacteria have been discovered and used for food preservation (Cortés‐Zavaleta, López‐Malo, Hernández‐Mendoza, & García, 2014; Kecerová, Pristas, & Javorský, 2004). A novel antimicrobial against *Bacillus* spp. produced by *L. plantarum* JLA‑9 was segregated and studied in its application fields (Zhao et al., 2016). Phenyllactic acid produced by lactic acid bacteria is a potential natural food preserver and preservative (Valerio, Lavermicocca, Pascale, & Visconti, 2004). In fact, antimicrobial activity of* L. plantarum* is mainly associated with the organic acids production and lowering pH of environment as indicated by references (Ołdak, Zielińska, Rzepkowska, & Kołożyn‐Krajewska, 2017). Some *L. plantarum* strains producing large amounts of organic acid were added to many fermented foods as a preservative (Li et al., 2016). This preservation method is very suitable for acid‐proof fermented food. Organic acids and their salts are considered weak acids, meaning they do not entirely dissociate in water but do so in a pH‐dependent manner. Organic acids are deemed to affect microbial activity by two primary mechanisms: cytoplasmic acidification with subsequent uncoupling of energy production and accumulation of the dissociated acid anion to toxic levels (Taylor et al., 2012). The antimicrobial mechanism of different organic acids is equally inconsistent. Therefore, different organic acids have different antimicrobial activities. The study showed that different kinds of lactic acid bacteria produce different types of organic acids, and even some of them produced more acetic acid than lactic acid (Rowland et al., 2010; Tejerosariñena, Barlow, Costabile, Gibson, & Rowland, 2012). In addition, *Lactobacillus* strains usually produce more than one organic acid, and the difference in the proportion of organic acids may be the reason why lactic acid bacteria's antimicrobial activities were inconsistent (Thu, Foo, Loh, & Bejo, 2013). However, the researches on the cooperation of different organic acids with a certain proportion have not been carried out deeply before.

In this study, the first aim was to screen *L. plantarum* strains with strong antimicrobial activity from traditional dairy products in Xinjiang. Then, the content of organic acids in the fermentation broths of *L. plantarum* strains was analyzed to study the relationship between organic acid content and its antimicrobial ability. Therefore, the second purpose was to find the suitable organic acid mixture with strong antimicrobial activity.

## 2 MATERIALS AND METHODS

### 2.1 Isolation and identification of lactic acid bacteria

Five samples of traditional dairy products, handmade by herdsmen in Xinjiang were collected. Each time 1 g of dairy products was transferred aseptically into 10 ml physiological saline and homogenized thoroughly. Samples were serially diluted in physiological saline. 0.1 ml samples of dilutions ranging between 10^−3^and 10^−7^were plated in duplicate on the surface of Ma–Rogosa–Sharpe (MRS Solarbio) agar with supplemented with 0.0025% of bromocresol green (Macklin) (Fhoula et al., 2013). The plates were incubated at 30°C for 48 hr. The different colonies of acid‐producing bacteria were picked on MRS agar by a yellow zone in the media around each colony.

These colonies were initially subjected to Gram stain assay. Gram‐positive strains were transferred to genetic identification using PCR method and 16S rDNA sequencing. The genomic DNA of the LAB strains was extracted using DNA Extraction Kit (TransGen Biotech) following the manufacture's protocol. 16S rDNA gene‐specific fragment primers for identification of strains were prepared in Sangon Biotech. The primers couple was 27F/1492R (5′‐AGA GTT TGA TCC TGG CTC AG‐3′/5′‐GGT TAC CTT GTT ACG ACT T‐3′). PCR conditions consisted of 30 cycles (1 min at 94°C, 45 s at 54°C, 2 min at 72°C) plus one additional cycle at 72°C for 7 min as a final chain elongation (Pennacchia et al., 2004). PCR products were separated from agarose gel (1.5% w/v), and the amplified fragments were visualized by staining with ethidium bromide under UV light. Their 16S rRNA gene sequences were executed with the Majorbio technique, and the BLAST program was used for sequence comparison (Kullen, Sanozkydawes, Crowell, & Klaenhammer, 2010).

### 2.2 Bacterial strains and growth conditions

Bacterial cultures were stored at −80°C with 20% glycerol (w/v). All *L. plantarum* strains were growth in MRS broth at 30°C under anaerobic conditions.

Indicator strains used for assessment of antimicrobial activity were *S. aureus* ATCC12600 (*S. aureus*), *E. coli* ATCC35128 (*E. coli*), and *Salmonella* ASI.1174 (*Salmonella*). All pathogens were grown in Luria–Bertani broth (LB, Oxoid) at 37°C, and solid medium was prepared by adding 1.5% agar to the broth media.

### 2.3 Antimicrobial properties of *L. plantarum* strains

The antimicrobial activity was evaluated by well diffusion method (Bonev, Hooper, & Parisot, 2008; Yang et al., 2017). The cultures of *L. plantarum* strains were grown in MRS broth (pH 6.5) for 24 hr to measure the antimicrobial properties of extracellular metabolites of *L. plantarum* strains. So the cultures were centrifuged (8,000 *g* for 10 min, 4°C) and filter sterilized through 0.22‐µm hydrophilic Durapore PVDF membrane (Nylon; RephiLe Bioscience). The cell‐free supernatant was recovered and tested for antimicrobial properties. To investigate the chemical nature of the potentially inhibitory substances secreted by each *L. plantarum* strain, showing the antagonistic effects, the supernatants were submitted to different treatments according to Herreros et al. (2005). First of all, the supernatants were heated at 100°C for 5 min and neutralized with 1 M NaOH to pH 6.5, in order to judge the antibacterial contribution of organic acids. Then, the neutralized supernatants were treated with catalase (1 mg/ml; Sigma‐Aldrich Corporation) at 37°C for 1 hr, and then pH of the supernatants were adjusted back to the original state, in order to rule out inhibiting effects due to hydrogen peroxide. At last, the neutralized supernatants were digested at 37°C for 2 hr with different proteases, that is, proteinase K (1 mg/ml), trypsin (1 mg/ml), and pepsin (1 mg/ml), in order to determine whether strains can produce antimicrobial peptides.

Pathogenic bacteria were grown overnight and diluted into physiological saline. Sterile Petri dishes were poured with LB agar and inoculated with 500 µl cultures of each indicator strain severally (concentration 7 log CFU/ml). After that Oxford plates were lightly placed on the surface of the LB agar plates and all treated supernatants were collected and 200 µl of each was used to fill Oxford plates previously on LB agar plates. The plates were incubated for 2 hr at 4°C in order to permit supernatants diffusion onto LB agar. All plates were incubated at 37°C for 24 hr, and then, the diameters of inhibition zones around the Oxford plates were measured. Antimicrobial activity (*x*) was calculated as follows: *x* = *D*−*d*, where *D* is the inhibition zone diameter and *d* is the Oxford plate diameter. The value of *x* represents the antimicrobial activity.

### 2.4 HPLC analysis of organic acids in cell‐free supernatants

Comparing the antimicrobial activities of various *L. plantarum* strains, three *L. plantarum* strains were selected to determine the organic acid content of their fermentation broths. According to the pH curves of *L. plantarum* strains, it can be found that and the pH of *L. plantarum* strains reaches 3.80 ± 0.05 and tends to stabilize at 24 hr. From this, we can speculate that *L. plantarum* strains reach the end of the log‐phase and the number of newly formed cells is equal to the number of dying cells after 24 hr. Therefore, the fermentation broths fermented for 24 hr were used as the sample to be tested. Ten milliliter of the fermentation broths obtained was centrifuged (8,000 *g* for 10 min, 4°C) to obtain cell‐free supernatants. Then, cell‐free supernatants were added to 1 ml of ammonium dihydrogen phosphate buffer with 3% methanol and were homogenized and centrifuged (14,000 *g* for 15 min, 4°C) to fully precipitate protein. Supernatants were 0.22‐μm‐filtered (Nylon; RephiLe Bioscience) into HPLC amber vials. For controls, unfermented MRS broth was treated under the same conditions.

Seven common organic acids were selected for the determination: oxalic acid, tartaric acid, malic acid, lactic acid, citric acid, acetic acid, and succinic acid. The reagents used were analytically pure, and tartaric acid was L (+), and malic acid was L (−). First, the standard curves of seven organic acids were identified separately. At the same time, the retention time of eight organic acids was determined.

A Shimadzu Nexera LC system with a photodiode array detector (SPD‐M20A) was utilized to detect and quantify the organic acids. The chromatographic separation was performed on a C_18_ column (250 × 4.6 mm I.D., 5 μm; Teknokroma). The organic acids were eluted using H_2_O with 11.5% ammonium dihydrogen phosphate (solvent A) and methanol (solvent B). Both solvents were 0.22‐μm‐filtered and degassed before use. Isocratic elution: 97% solvent A and 3% solvent B. The flow rate was set to 0.7 ml/min, the temperature was set to 25°C, and a volume of 10 μl was injected. Organic acids were detected at a UV wavelength of 210 nm.

### 2.5 Antimicrobial properties of organic acids produced by *L. plantarum* strains

There were five kinds of organic acids detected in the fermentation broths of three *L. plantarum* strains, which are lactic acid, acetic acid, tartaric acid, malic acid, and citric acid by HPLC analysis. Using five organic acids, respectively, the pH of MRS broth was adjusted to 3.80 ± 0.05. Then, the pH‐adjusted MRS broths were used to measure the antimicrobial activity using well diffusion method. MRS broths pH adjustment using HCl as control was treated under the same conditions.

According to the content of different organic acids in the fermentation broths, utilizing exogenous organic acids, antimicrobial agents containing organic acids were configured. The antimicrobial agents corresponding to fermentation broths of *L. plantarum* P1, *L. plantarum* S11, and *L. plantarum* M7 were P1‐1, S11‐1, and M7‐1. Then, these antimicrobial agents were centrifuged (8,000 *g* for 10 min, 4°C) and were 0.22‐μm‐filtered (Nylon; RephiLe Bioscience) to remove bacteria. Then, these antimicrobial agents were used to measure the antimicrobial activity using well diffusion method.

### 2.6 Statistics analysis

All experiments were performed three times. The results were subjected to Student's *t* test for the significant difference (*p* < 0.05) by GraphPad Prism (version 7.01).

## 3 RESULTS AND DISCUSSION

### 3.1 Isolation and identification of lactic acid bacteria

The acid‐producing indicator plate was used to screen single colonies with strong acid production ability. Ninety‐six strains were isolated on MRS agar from five kinds of traditional dairy products, depending on the size of the yellow area around a single colony on the MRS plate. Twenty‐six isolates (Table 1) were identified as rod‐shaped Gram‐positive and catalase‐negative bacteria, and all strains selected for the study demonstrated 98%–100% similarity to *L. plantarum* for 16S rDNA sequence.

**Table 1:** Results of strains screening and identification

Source	Strain	Acid production capacity (size of the yellow area)^a^	Gram (+/−)	Species
Kumis	K1	+	+	*Enterococcus faecium*
Milk thistle	G4	+	+	*Lactobacillus plantarum*
Yogurt	S1	++	+	*L. plantarum*
	S2	++	+	*L. plantarum*
	S4	+	+	*L. plantarum*
	S5	+	+	*L. plantarum*
	S6	++	+	*L. plantarum*
	S7	+	+	*L. plantarum*
	S8	++	+	*L. plantarum*
	S10	++	+	*L. plantarum*
	S11	+++	+	*L. plantarum*
	S12	+	+	*L. plantarum*
	S13	++	+	*L. plantarum*
	S21	+	+	*L. plantarum*
	S24	+	+	*L. plantarum*
Fermentation of millet	M7	++	+	*L. plantarum*
	M13	+	+	*L. plantarum*
	M14	+	+	*L. plantarum*
	M15	++	+	*L. plantarum*
	M17	++	+	*L. plantarum*
	M22	+	+	*L. plantarum*
Urum	P1	+++	+	*L. plantarum*
	P2	+	+	*L. plantarum*
	P5	+	+	*L. plantarum*
	P8	++	+	*L. plantarum*
	P16	+	+	*L. plantarum*
	P21	++	+	*L. plantarum*

a Acid production capacity: + weak ++ medium +++ strong.

### 3.2 Screening of strains with strong antimicrobial activity

**Figure 1:**
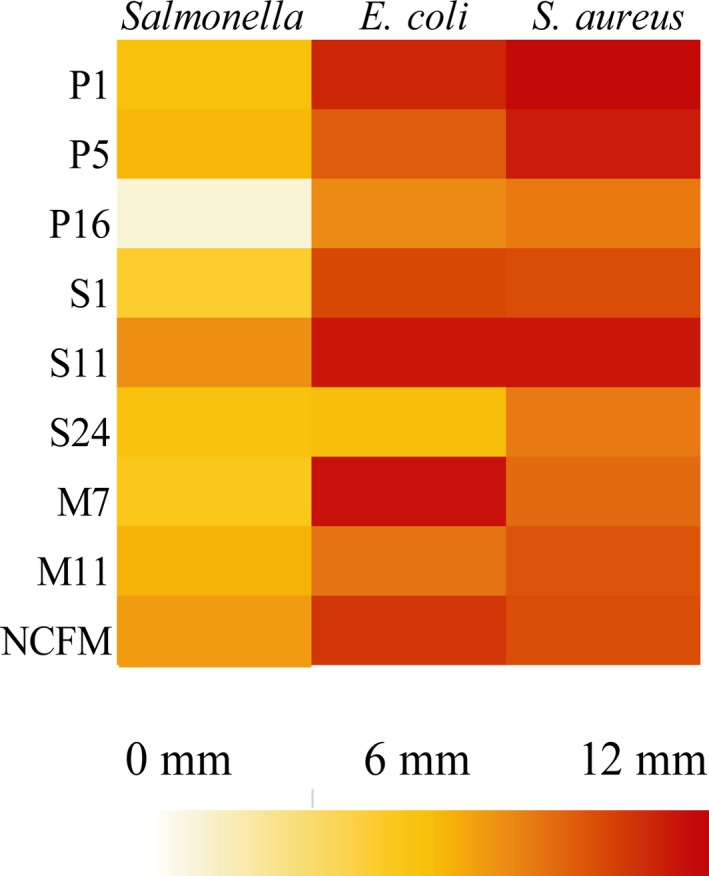
Inhibition zones map. Eight *Lactobacillus plantarum* strains and *Lactobacillus acidophilus* NCFM against three common pathogens. *S. aureus*: *S. aureus* ATCC12600 as indicator; *E. coli*: *E. coli* ATCC35128 as indicator; *Salmonella*: *Salmonella* ASI.1174 as indicator. Data are the mean ± *SD* of at least three independent experiments

The antimicrobial activity of nine strains (Table 2) was evaluated by well diffusion method. Obvious inhibition zone on the plate was observed, and the inhibition zone diameter ranged from 10 to 20 mm, which represents the strength of antimicrobial activity (Figure S1). Three common pathogens (Table 3) were used to assess the antimicrobial potential (Figure 1). Fermentation broths of different strains had different antimicrobial activities. All tested strains have antimicrobial activity against *S. aureus*, and antimicrobial activity (*x*) of *L. plantarum* P1 was the highest (11.7 mm). On the other hand, fermentation broths of *L. plantarum* M7 and *L. plantarum* S11 display high level of antimicrobial activity against *E. coli*, and *x* reached 11.4 and 11.2 mm separately. However, all the strains did not show the excellent ability to inhibit the proliferation of *Salmonella*, and *L. plantarum* S11 with the highest antimicrobial activity only reached 7.5 mm. After preliminary screening, *L. plantarum* P1, *L. plantarum* S11 and *L. plantarum* M7 were selected for the further analysis.

**Table 2:** *Lactobacillus* used in this work

Strain	Original source	Identification by 16S rRNA
P1	Urum	*Lactobacillus plantarum*
P5	Urum	*L. plantarum*
P16	Urum	*L. plantarum*
S1	Yogurt	*L. plantarum*
S11	Yogurt	*L. plantarum*
S24	Yogurt	*L. plantarum*
M7	Fermentation of millet	*L. plantarum*
M11	Fermentation of millet	*L. plantarum*
NCFM	DuPont	*Lactobacillus acidophilus*

**Table 3:** Pathogenic bacteria used in this work

Strain	Original source	Identification by 16S rRNA
ATCC12600	Saved in our lab	*Staphylococcus aureus*
ATCC35128	Saved in our lab	*Escherichia coli*
ASI.1174	Saved in our lab	*Salmonella typhimurium*

### 3.3 Analysis of antimicrobial substrate in fermentation broths of *L. plantarum* strains

**Figure 2:**
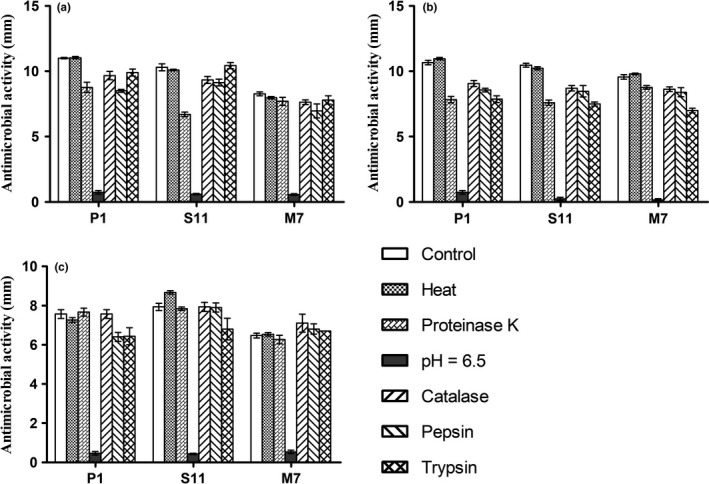
Changes of antimicrobial activity of fermentation broths of three *Lactobacillus plantarum* strains after various treatments. Control: Untreated fermentation broth; heat: 100°C heat treatment; proteinase K: the fermentation broth treated by proteinase K; pH = 6.5: the fermentation broth with pH adjusted to 6.5; catalase: the fermentation broth treated by catalase; pepsin: the fermentation broth treated by pepsin; trypsin: the fermentation broth treated by trypsin. (a) *S. aureus* ATCC12600 as indicator; (b) *E. coli* ATCC35128 as indicator; (c) *Salmonella* ASI.1174 as indicator. Data are the mean ± *SD* of at least three independent experiments

Lactic acid bacteria are a class of nonspore, Gram‐positive bacteria, whose principal common characteristic is fermenting sugars into organic acids. The decrease in pH can greatly inhibit the growth of other bacteria. Moreover, some other studies have also found that H_2_O_2_ produced during the metabolic process can inhibit bacteria (Charlier, Cretenet, Even, Loir, & Loir, 2009). On the other hand, some lactic acid bacteria can produce bacteriocins and bacteriocin‐like compounds to inhibit pathogens (Zhao et al., 2016). In this study, fermentation broths of *L. plantarum* strains (P1, S11, and M7) were carried out with five treatments to identify the major antimicrobial substrate. As shown in Figure 2, the antimicrobial activities against three pathogenic bacteria were not changed after heating and catalase treatment. Antimicrobial activities (*x*) of *L. plantarum* S11 and *L. plantarum* P1 slightly decreased after treated with proteinase K, pepsin, and trypsin, respectively. The proteinase K treatment reduced the antimicrobial activity of *L. plantarum* S11 against *S. aureus* from 10.2 to 5.6 mm (Figure 2a). On the contrary, pepsin and trypsin had no effect on the antimicrobial activity of fermentation broths. Most important of all, the inhibition zone diameter of the three fermentation broths all dropped below 1 mm after adjusting the fermentation broth pH from 3.80 to 6.50.

This phenomenon indicated that the antimicrobial activity of these three strains is due primarily to organic acids. Other than that, there was no heat‐sensitive antimicrobial substance in fermentation broths, such as macromolecule protein. Three kinds of *L. plantarum* strains (P1, S11, and M7) did not produce hydrogen peroxide with antimicrobial activity during fermentation. According to the effect of protease on the antimicrobial activity of fermentation broth, there may be polypeptide antimicrobial substances in fermentation broths of *L. plantarum* P1 and *L. plantarum* S11.

### 3.4 Common organic acids in fermentation broths of three *L. plantarum* strains

**Figure 3:**
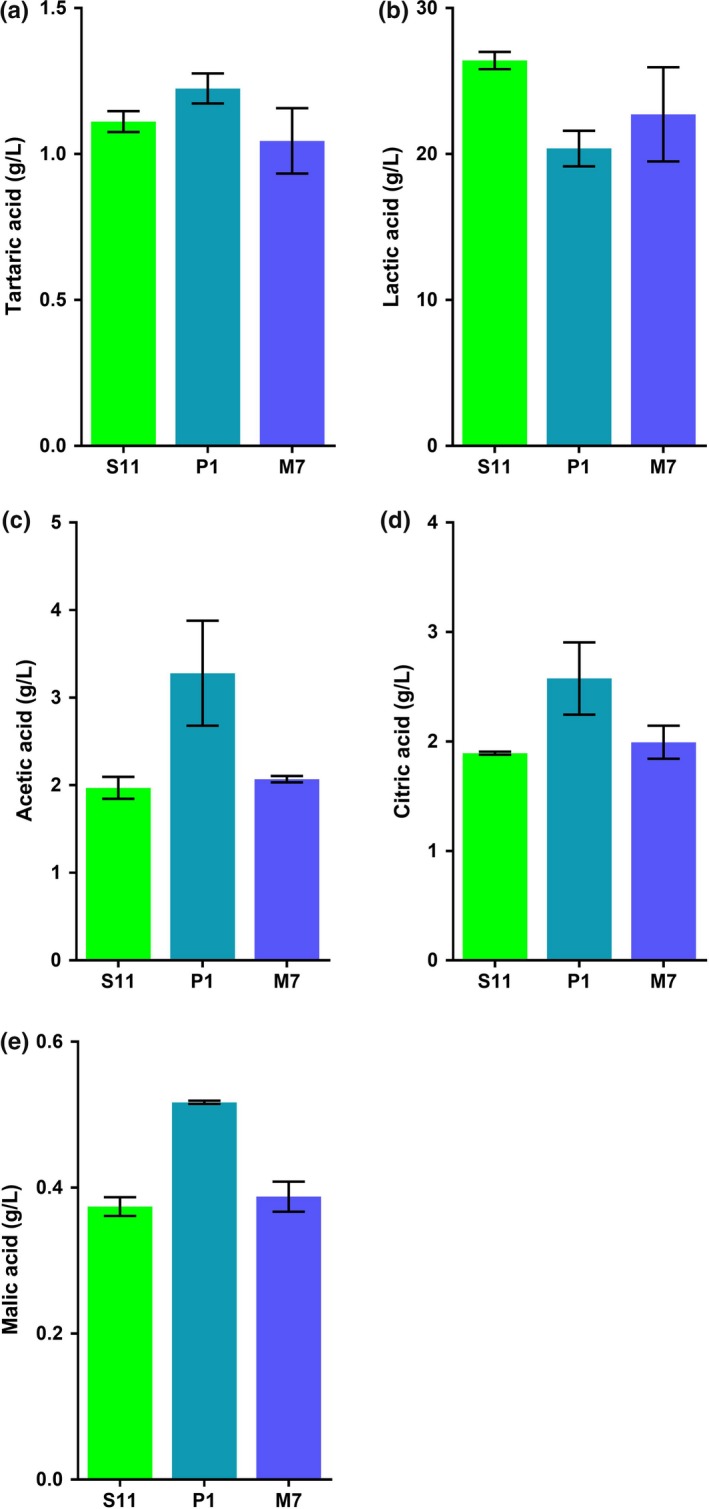
Analysis of organic acids in fermentation broths of three *Lactobacillus plantarum* strains by HPLC. (a) Analysis of tartaric acid in three fermentation broths; (b) analysis of lactic acid in three fermentation broths; (c) analysis of acetic acid in three fermentation broths; (d) analysis of citric acid in three fermentation broths; (e) analysis of malic acid in three fermentation broths. Data are the mean ± *SD* of at least three independent experiments

*Lactobacillus plantarum* strains can produce a variety of organic acids, mainly lactic acid. In point of metabolite products of *L. plantarum*, the main organic acids which own antimicrobial behavior are the acetic acids and lactic (Zalán, Hudáček, Štětina, Chumchalová, & Halász, 2010). In addition, other common organic acids such as tartaric acid and citric acid may also own antimicrobial activity. In our study, tartaric acid, malic acid, lactic acid, citric acid, and acetic acid were selected for the determination. According to pH curves of three *L. plantarum* strains in MRS, the pH of fermentation broths stabilizes at 3.80 ± 0.05 after 24 hr. Five kinds of organic acids were detected in the fermentation broths of these three strains by HPLC analysis (Figure S2). As shown in Figure 3, the organic acid produced by *L. plantarum* strains (P1, S11, and M7) was mainly lactic acid which was the highest in *L. plantarum* S11 (26.4 g/L). Compared to *L. plantarum* S11 and *L. plantarum* M7, *L. plantarum* P1 was detected with the highest level of acetic (3.3 g/L) and lactic acid (2.6 g/L), respectively. Beyond that, a small amount of tartaric acid and malic acid were also detected in all fermentation broths.

Compared with other organic acids, the content of lactic acid is the highest and it is the main substance that decreases the pH of the broth. The decrease in pH plays a certain inhibitory effect on the pathogenic bacteria growth. However, other organic acids also contribute to the antimicrobial activity. Therefore, the antimicrobial activity of these five organic acids was mainly studied in this study.

### 3.5 Configuration of antimicrobial agents and determination of their antimicrobial activities

**Figure 4:**
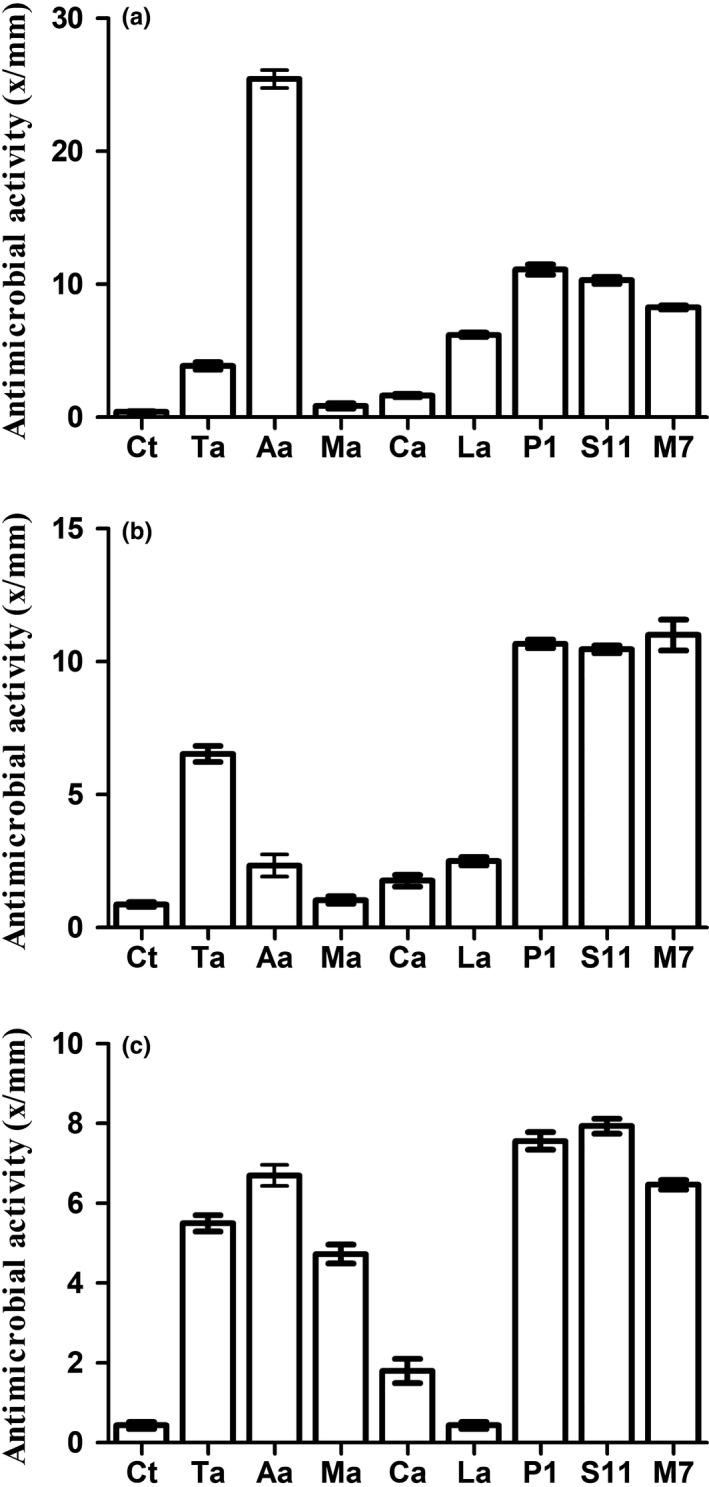
Comparison of the antimicrobial activity of five organic acids and fermentation broths at the same pH value. Ct: MRS (pH 6.2). (a) *S. aureus* ATCC12600 as indicator; (b) *E. coli* ATCC35128 as indicator; (c) *Salmonella* ASI.1174 as indicator. Data are the mean ± *SD* of at least three independent experiments

The antimicrobial activities of these five organic acids are presented in Figure 4. Since the pH of three *L. plantarum* strains stabilized at 3.8 ± 0.05 after 24 hr, pH of MRS mediums was adjusted to 3.80 ± 0.05 using exogenous five organic acids respectively. Then, the antimicrobial activities of these mediums were measured by well diffusion method. When Gram‐positive *S. aureus* was the indicator, the antimicrobial activity of acetic acid was far higher than that of *L. plantarum* strains (P1, S11 and M7), reaching 26.2 mm (Figure 4a). However, the antimicrobial activity of the other four organic acids is lower than that of the fermentation broths. When using *E. coli* and *Salmonella* as indicator bacteria, the antimicrobial activities of three *L. plantarum* strains (P1, S11, and M7) were higher than those of five organic acids (Figure 4b,c).

Food preservation with organic acids is a very effective method. Fermentation broths of *L. plantarum* strains (P1, S11, and M7) contained a variety of organic acids, and their antimicrobial activities against *E. coli* and *Salmonella* were stronger than any single organic acid at the same pH value. Therefore, we could speculate that the synergistic inhibition of different organic acids may be stronger than that of a single organic acid. Inspired by this, a new type of antimicrobial agent can be configured by mixing common organic acids in different proportions according to the broths.

**Figure 5:**
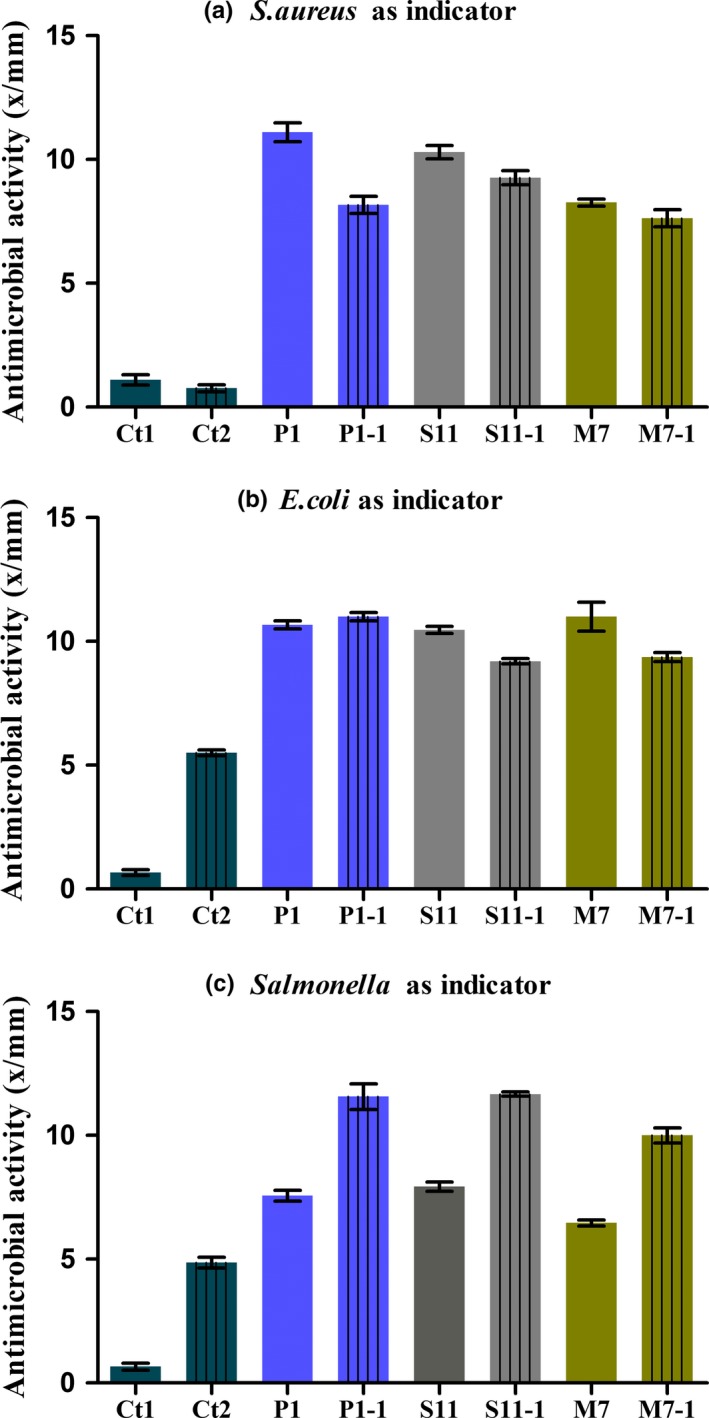
Comparison of antimicrobial activity of the fermentation liquid and exogenous organic acid mixed solution of three *Lactobacillus plantarum* strains. Ct1: Untreated MRS; Ct2: MRS with pH adjusted with hydrochloric acid. Data are the mean ± *SD* of at least three independent experiments

Depending on the proportion of organic acids shown in Figure 3, antimicrobial agents P1‐1, S11‐1, and M7‐1 were configured by five organic acids. The antimicrobial effect was tested, and the results are shown in Figure 5. The pH of the antimicrobial agents formulated with the exogenous organic acid was kept at 3.80, which was consistent with the fermentation broth. Using *S. aureus* as an indicator, the antimicrobial activity of P1‐1 is 4 mm which was smaller than that of *L. plantarum* P1 fermentation broth (Figure 5a). The antimicrobial activity of S11‐1 and M7‐1 is consistent with the corresponding fermentation broths, and among them, S11‐1 had the highest antimicrobial activity, reaching 9.2 mm. Using *E. coli* as an indicator, fermentation broth of *L. plantarum* P1 and P1‐1 had the same antimicrobial activity, reaching 12.2 mm, and the antimicrobial activities of S11‐1 and M7‐1 are about 2 mm which were smaller than the fermentation ones (Figure 5b). Using *Salmonella* as an indicator, the antimicrobial activities of three antimicrobial agents (P1‐1, S11‐1, and M7‐1) are far higher than those of fermentation broths (Figure 5c).

Compared with *L. plantarum* strains (P1, S11, and M7), the antimicrobial activities of antimicrobial agents (P1‐1, S11‐1, and M7‐1) are relatively higher. Making a new antimicrobial agent by mixing organic acids is a potential method for preserving foods effectively. In addition, for different pathogenic bacteria, it can be targeted by adjusting the proportion of organic acids. In conclusion, antimicrobial agents with outstanding antimicrobial effects can be configured by mixing a certain proportion of common organic acids. The antimicrobial activity could be increasing by adjusting the organic acids proportions for the different pathogenic bacteria.

## 4 CONCLUSIONS

The results of this study showed that three *L. plantarum* strains (P1, S11, and M7) isolated from Xinjiang traditional dairy products showed strong antimicrobial activities against indicator strains. Moreover, organic acids played a key role in antimicrobial substances in fermentation broths. Five common organic acids were found in the fermentation broth, and the proportion of organic acids in the *L. plantarum* strains (P1, S11, and M7) fermentation broths was different, matching their different antimicrobial effects. Thus, depending on the proportion of organic acids in the three fermentation broths, five organic acids were mixed to make three artificial antimicrobial agents (P1‐1, S11‐1, and M7‐1). We found that the antimicrobial activity of the three antimicrobial agents against pathogenic bacteria was strong. At the same pH value, the antimicrobial activity of mixed organic acids is sometimes stronger than that of single organic acids. In addition, organic acid antimicrobial agents combined in different proportions can inhibit different pathogenic bacteria. Therefore, it is a simple and effective way to develop new antimicrobial agents through the cooperation of different organic acids.

## CONFLICT OF INTEREST

No conflict of interest declared.

## ETHICAL STATEMENT

This study does not involve any human or animal testing.

## INFORMED CONSENT

Written informed consent was obtained from all study participants.
