# Supplementary figures and images for: Characterization of antimicrobial activity of three Lactobacillus plantarum strains isolated from Chinese traditional dairy food
Source: Food Sci Nutr. 2019 Apr 29;7(6):1997–2005. doi: 10.1002/fsn3.1025 (PMC6593389; doi:10.1002/fsn3.1025)

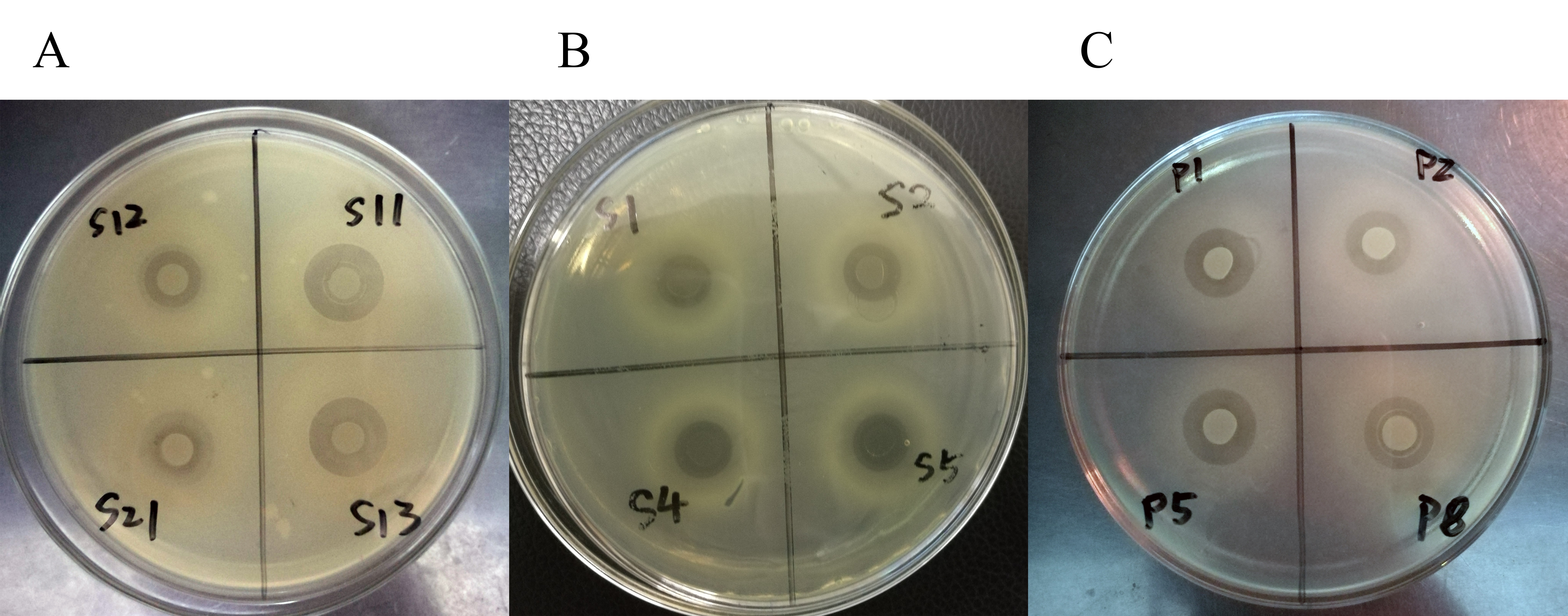

Supplement: Supplementary file 1 [file FSN3-7-1997-s001.jpg]

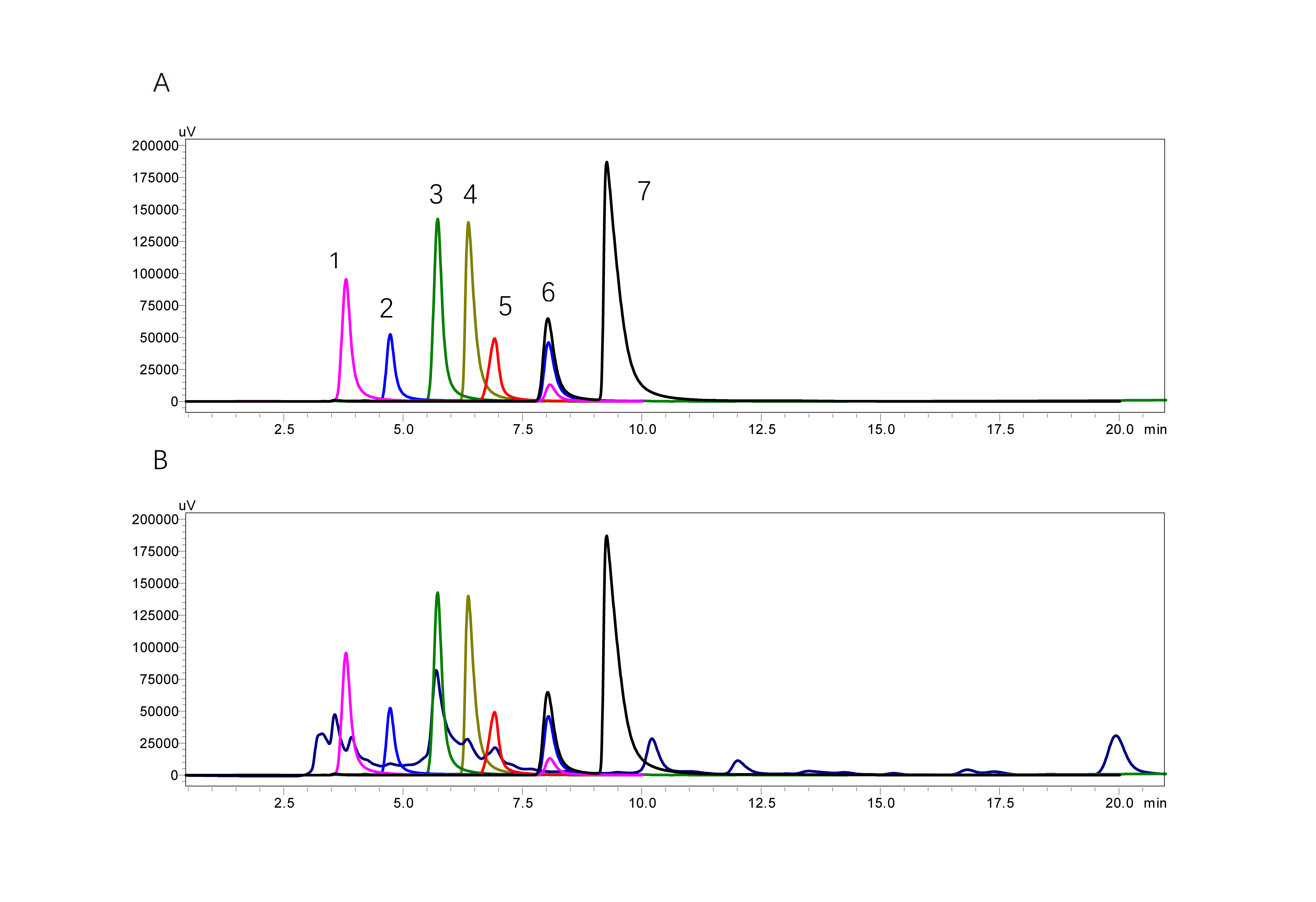

Supplement: Supplementary file 2 [file FSN3-7-1997-s002.tif]
