# Supplementary material for: Characterization of antimicrobial activity of three Lactobacillus plantarum strains isolated from Chinese traditional dairy food
Source: Food Sci Nutr. 2019 Apr 29;7(6):1997–2005. doi: 10.1002/fsn3.1025 (PMC6593389; doi:10.1002/fsn3.1025)
Supplement: Supplementary file 3 [file FSN3-7-1997-s003.docx]

Fig. S1 The inhibition zones diameter of selected strains against three common pathogens. A: *S. aureus* ATCC12600; B: *E. coli* ATCC35128; C: *Salmonella* ASI.1174.

Fig. S2 HPLC chromatogram of common organic acids. 1:tartaric acid; 2: Malic acid; 3:Lactic acid; 4: Acetic acid; 5:Citric acid; 6:Solvent; 7:Succinic acid. A: HPLC chromatogram of 6 organic acid standards; B: HPLC chromatogram of the sample.
